# Recent Advances in Reversible Thermochromic Materials for Smart Textiles: A Review

**DOI:** 10.3390/ma19040742

**Published:** 2026-02-14

**Authors:** Qiucheng Lu, Xu Wang, Xiaohui Zhao, Ziqiang Bi, Hailin Li, Yuqing Liu

**Affiliations:** 1 School of Arts and Innovation Design, Suzhou City University, Suzhou 215104, China; 2 School of Optical and Electronic Information, Suzhou City University, Suzhou 215104, China; 3 College of Textile and Clothing Engineering, Soochow University, Suzhou 215123, China

**Keywords:** reversible thermochromic materials, smart textiles, textile integration technologies, thermochromic applications, color-changing properties

## Abstract

**Highlights:**

**What are the main findings?**

- Summarizes characteristics and recent advances of four types of reversible thermochromic materials: organic, liquid crystal, inorganic, and photonic crystal.
- Details textile integration technologies including microencapsulation, printing/dyeing, and fiber fabrication, with latest research progress.
- Analyzes applications of smart textiles in anti-counterfeiting, temperature regulation, and aesthetic enhancement.
- Addresses current challenges (stability, wash durability, color sensitivity) and proposes potential development directions.

**What are the implications of the main findings?**

- Provides a theoretical foundation and technical guidance for the design and development of reversible thermochromic smart textiles.
- Promotes deep integration of traditional textile craftsmanship (e.g., Kesi) with modern thermochromic technology.
- Facilitates the advancement of multifunctional, intelligent, and environment-adaptive smart textiles.
- Offers new perspectives for solving key technical bottlenecks in practical applications of thermochromic materials.

**Abstract:**

Reversible thermochromic materials change color in response to temperature variations and hold significant potential in smart textiles. Their reversible color-changing property not only offers temperature indication and enhances textile performance but also promotes smart textile development. This is achieved by improving the intelligence, multifunctionality, and environmental adaptability of textiles. This review summarizes the characteristics and recent advancements of reversible thermochromic materials, including leuco dye-based organic systems and other organic, liquid crystal (LC), inorganic, and photonic crystal (PC) types. It emphasizes recent progress in integrating these materials into textiles through techniques such as microencapsulation, printing and dyeing, and fiber fabrication. Furthermore, this review systematically examines applications of reversible thermochromic materials in smart textiles, covering areas such as anti-counterfeiting, temperature-sensitive regulation, and aesthetic enhancement. Current challenges, including limited stability, inadequate wash durability, and low color sensitivity, are also addressed, alongside potential development directions. The aim of this review is to provide a theoretical foundation and technical guidance for designing and developing reversible thermochromic smart textiles.

## 1. Introduction

Driven by the growing demand for functional and diverse daily products [1,2,3], smart textiles have emerged as an important research field. They represent the integration of textile engineering with electronics [4,5] information technology [6], biology [7], medicine [8], and renewable energy [9]. These advanced materials can detect and respond to changes in the human body and environment while maintaining the inherent aesthetic and technical features of traditional textiles [10]. Among the main categories of smart textiles, including shape memory textiles and waterproof breathable fabrics, reversible thermochromic materials have attracted increasing attention [11,12,13,14,15,16,17].

As a class of temperature-sensitive functional materials, thermochromic materials change in color with temperature variations. They are either irreversible or reversible depending on their color-changing behavior [18]. During color changes, irreversible thermochromic materials undergo permanent physical or chemical structural changes. However, they are incapable of cyclic recovery in color [19]. In contrast, reversible thermochromic materials are capable of multiple and cyclic color changes when the temperature rises and falls repeatedly. This is enabled by the reversible physical or chemical processes within the material system [20]. The reversible thermochromic materials discussed in this paper and suitable for the textile industry possess multiple functional properties. They are susceptible to physical transformations or structural rearrangements in response to the changes in ambient temperature. With such structural changes altering their spectral properties, they ultimately exhibit observable and cyclically recoverable color changes [21,22,23,24].

**Figure 1:**
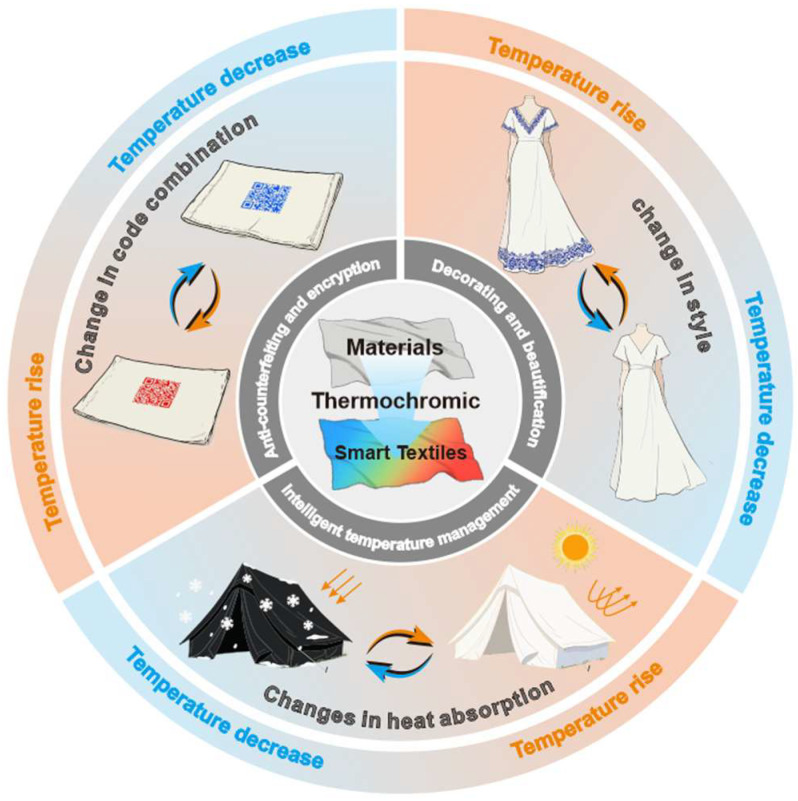
The illustrated representation of reversible thermochromic smart textiles in use [21,36,37,38].

A significant challenge is ensuring thermal sensitivity, reversibility, and stability in textile applications [25,26,27]. Recent studies have emphasized embedding thermochromic microcapsules containing leuco dyes into polymer coatings, fibers, or fabric inks [28,29]. Concurrently, research on inorganic thermochromic pigments, such as vanadium dioxide (VO_2_), has shown excellent thermal and chemical stability [30,31]. Recent advances in anti-counterfeiting [32,33,34,35,36], decoration [37], and thermal management [38] (Figure 1) have been achieved. These developments have further increased interest in reversible thermochromic smart textiles. Although research and development in this area have expanded in recent years, systematic reviews remain limited. Notably, the incorporation of reversible thermochromic materials into traditional textile techniques such as Kesi is still at an early stage.

This review systematically examines reversible thermochromic materials and their integration into smart textiles, focusing on current developments and challenges. It discusses applications in anti-counterfeiting, decoration, and thermal management, highlighting their value and potential. Furthermore, this review aims to promote the deep integration of traditional craftsmanship and modern technology. By addressing a research gap and providing foundational knowledge for combining thermochromic materials with traditional methods, this work offers new perspectives for designing the next generation of smart textiles.

## 2. Reversible Thermochromic Materials

Reversible thermochromic textile materials are substances that undergo physical transformations or structural rearrangements in response to changes in ambient temperature. These transformations alter their spectral properties including visible light absorption, reflection and scattering characteristics in the visible light range, resulting in observable color changes [39].

### 2.1. Organic Reversible Thermochromic Materials

**Figure 2:**
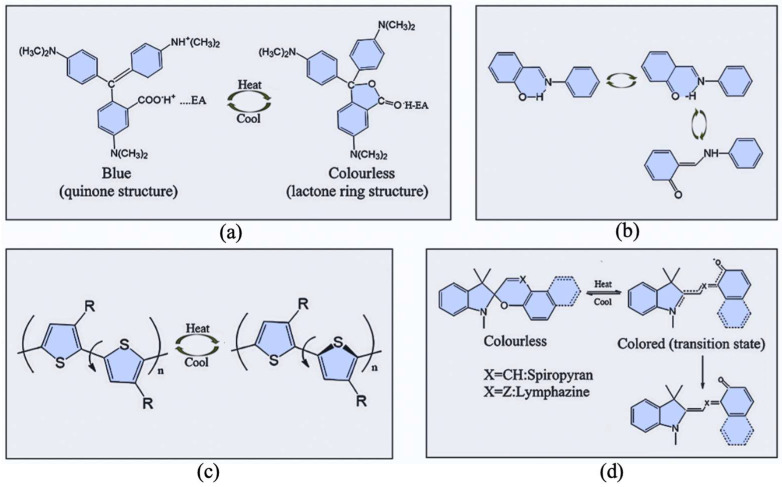
Structural changes in molecules during color change in organic thermochromic materials. (**a**) electron transfer process of crystal violet lactone, (**b**) keto-enol tautomerism of N-Salicylideneaniline, (**c**) conformational change in polythiophene, and (**d**) ring-opening mechanism of spiropyran [21,44,45].

Compared to inorganic and liquid crystal (LC) reversible thermochromic materials, organic reversible thermochromic materials offer advantages such as vivid colors, distinct color changes, and high temperature sensitivity. The color transition range can be precisely adjusted by modifying the dye structure, enabling a wide variety of colors including red, green, yellow, blue, and black [40,41,42,43]. The color change mechanisms in these materials involve intermolecular electron transfer, molecular conformational changes, crystal phase transitions, and molecular ring-opening, as illustrated in Figure 2.

Thermochromism driven by intermolecular electron transfer is predominantly found in organic thermochromic systems. This process typically involves a leuco dye, a developer, and a solvent [44,45]. The leuco dye acts as an electron donor and determines the initial color. After electron transfer, it undergoes structural changes, resulting in color variation. Common leuco dyes mainly include triarylmethanes, fluorans, Schiff bases, spiro compounds and other organic complexes [21]. The molecular structure of triarylmethanes is centered on a central carbon atom that is directly bonded to three aromatic groups. Fluorans are derivatives of xanthene-fused lactones. Schiff bases possess a typical molecular structure of R_1_R_2_C=NR_3_. Spiro compounds are structurally characterized by containing a spiro carbon that links two ring systems. The developer serves as an electron acceptor and consists of both inorganic and organic types. Common organic developers mainly include phenolic hydroxyl compounds and their derivatives, as well as carboxylic acids and their derivatives. Other typical developers include sulfonic acids, acid phosphates and their metal salts, triazines, and halogenated alcohols and their derivatives. The solvent (temperature modifier) is an organic compound that regulates temperature sensitivity. Its melting point largely determines the color-changing temperature of reversible thermochromic materials. Common solvents include higher fatty alcohols, thiols, ketones, phosphates, sulfonates, carboxylates, and sulfites.

**Figure 3:**
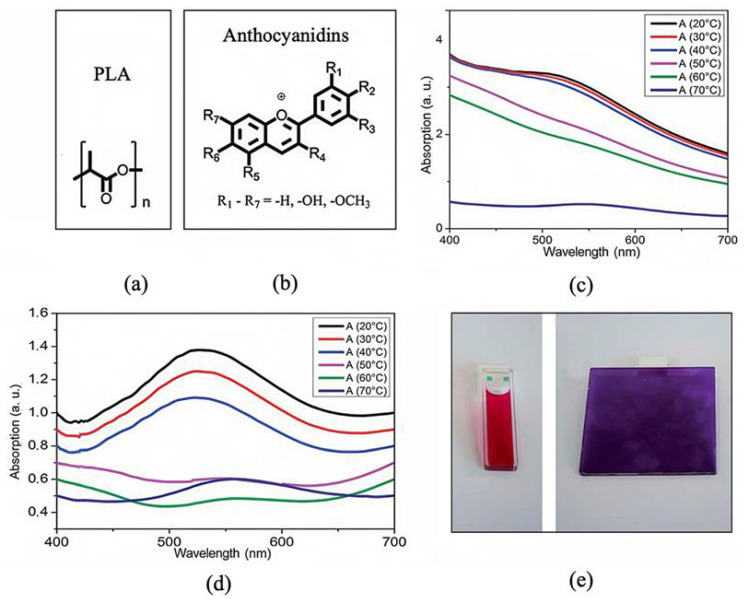
Molecular structures of PLA and anthocyanin, and temperature dependence of visible light absorption in PLA composites: (**a**) molecular structure of PLA, (**b**) molecular structure of anthocyanin, (**c**) original curves of PLA composites during heating, (**d**) slope-corrected curves of PLA composites during heating, and (**e**) color variation effect. (reused with permission, ref. [50] © 2013, Royal Society of Chemistry, RSC).

The preparation of organic reversible thermochromic composites involves three components, leuco dye, developer, and solvent, which require materials with good compatibility. The choice and combination of these components influence several key factors. These factors include the tunability of the color-changing temperature, flexibility in color matching, visibility of the color shift, and cost. Among the common selections, crystal violet lactone and bisphenol A (BPA) serve as the leuco dye and developer, respectively, due to their wide color range and stability [41,46,47]. Crystal violet lactone (Figure 3a), one of the earliest triarylmethane thermochromic dyes, changes color primarily through the reversible opening and closing of its internal lactone ring structure [48]. Mao Dongyu et al. combined crystal violet lactone, BPA, and 1-tetradecanol to form a thermochromic composite, determining an optimal component ratio of 1:40:70 through experiments. The resulting material exhibited a color-changing time of 42 s as well as excellent thermal and acid–base resistance [49]. Beyond this common system, researchers [36] have developed a reversible thermochromic material based on sulfones, using bromocresol purple (BCP) as the developer. The color-changing temperature of this material can be adjusted within 13–46 °C. In a heating–cooling cycle test, it maintained stable reversibility with no significant degradation of color parameters after 50 cycles. Experimental data show that targeted adjustment of the thermochromic points is achievable. This requires precise control over the thermochromic transition temperature. Such control is achieved by varying the ratio of long-chain alcohols to stearic acid. The alcohols used in this study are dodecanol, tetradecanol, hexadecanol, and octadecanol.

Lötzsch and colleagues [49,50,51,52] have focused on natural materials as substitutes for conventional thermochromic systems and identified anthocyanin as a naturally occurring organic compound. The interaction between poly(lactic acid) (PLA) and cyanidin chloride was systematically analyzed. As a result, a reversible and non-toxic thermochromic composite was successfully prepared, as shown in Figure 3b. This anthocyanin-based thermochromic PLA composite comprises PLA, cyanidin chloride, dodecyl gallate, and hexadecanoic acid. Upon heating from 20 °C to 70 °C, the first transition is the glass transition of the polymer matrix occurring between 40 and 50 °C, followed by the melting of hexadecanoic acid-rich domains at 60–70 °C (Figure 3c). Both transitions cause significant changes in light scattering. Figure 3d reveals that the thermochromic transition coincides with the glass transition temperature. The PLA matrix transitions from a glassy state to a highly elastic state at the glass transition temperature. This transition disrupts the hydrogen bonding interactions between PLA and anthocyanin, enhances the polarity and free volume of the microenvironment, and increases the mobility of dye molecules. These changes promote the transformation of anthocyanin from the neutral anhydrobase form to the anionic anhydrobase form, leading to a red shift in the absorption peak from 530 nm to 560 nm and realizing the visible color change from red to purple. The reversibility of the matrix phase transition and the stable dispersion of nano-sized hexadecanoic acid-rich domains ensure the reversibility and cycling stability of the thermochromic effect [50]. In the glassy state, the absorption maximum is near 530 nm, shifting to about 560 nm above the glass transition. As demonstrated in Figure 3e, the color changes from red to purple.

### 2.2. Reversible Thermochromic Materials Based on LCs

**Figure 4:**
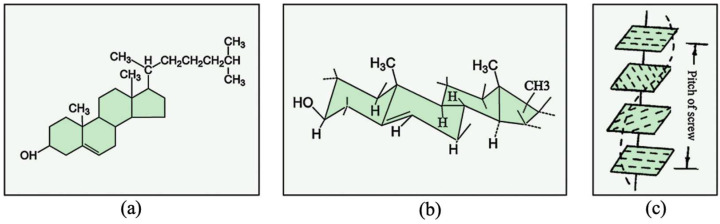
Several structures of cholesteric LCs: (**a**) structural formula, (**b**) configuration, and (**c**) helical structure [21,56].

LCs exhibit excellent stability and high sensitivity to temperature changes. However, their application is limited due to chemical sensitivity and relatively high costs. LCs are classified into three types based on molecular arrangement: smectic, nematic, and cholesteric. Cholesteric LCs are the most commonly used type [21,53,54,55]. Cholesteric LCs (Figure 4), also known as chiral nematic LCs, possess a periodic helical superstructure, classifying them as a type of “soft” PC. Reversible thermochromic LCs have been developed for textile printing and dyeing [56,57]. LC-based reversible thermochromic materials have several advantages. These include low color-changing temperature, adjustable temperature range, and high color sensitivity. However, they also have drawbacks, such as high cost and weak color-changing intensity [58]. LCs lack affinity for fibers, which limits their direct application in textiles. Therefore, they must be encapsulated into microcapsules and combined with polymeric binders for textile applications [59,60,61]. This process reduces the brightness and tactile quality of LC-based thermochromic products.

**Figure 5:**
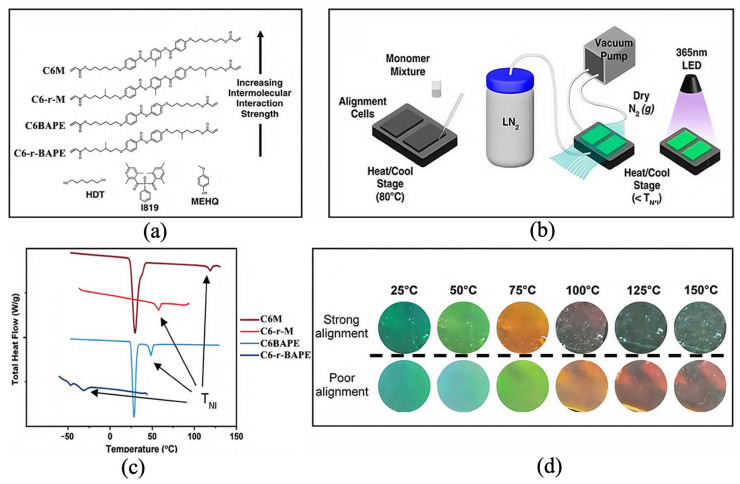
Tunable MDSC: (**a**) chemical structure of CLCE monomers, (**b**) schematic diagram of CLCE preparation method, (**c**) thermograms of pure components obtained by modulated differential scanning calorimetry, and (**d**) changes in strongly and weakly aligned cholesteric LCs at different temperatures. (reused with permission, ref. [65] © 2024, ACS Publications).

Cholesteric liquid crystalline elastomers (CLCEs) are typically synthesized through the copolymerization of chiral and achiral monomers. These monomers contain functional end groups [62,63,64]. Schlafmann et al. [65] demonstrated that tunable and switchable thermochromic effects can be obtained by adjusting the cross-linking density and LC composition of CLCEs. These adjustments allow for different thermochromic responses upon heating. Their study focused on CLCEs composed of two achiral liquid crystalline diacrylates (C6M and C6BAPE) and one chiral liquid crystalline diacrylate (C6-r-M or C6-r-BAPE), shown in Figure 5a,b. Modulated differential scanning calorimetry (MDSC) data in Figure 5c indicate that C6M and C6-r-M have the highest phase transition temperatures. This is attributed to their mesogenic cores, which are composed of 1, 4-bis(4-[n-acryloyloxybutoxy]benzoyloxy)-2-methylbenzene. The presence of two methyl groups on the aliphatic chain of C6-r-M creates chiral centers that influence mesogen packing and arrangement, lowering the nematic–isotropic transition temperature (T_NI_) and the crystallization temperature (T_C_) during cooling. C6BAPE contains a biaryl core structure, which weakens intermesogenic interactions, resulting in a reduced T_NI_. C6-r-BAPE exists as an isotropic liquid at room temperature, exhibiting a T_NI_ below room temperature (−31.7 °C) during cooling. Due to the weakened intermesogenic interactions caused by these monomers, the T_NI_ decreases, and the low birefringence in some CLCE formulations enables tuning of thermochromic reflection. By varying CLCE compositions, different thermochromic effects can be achieved, as shown in Figure 5d. Experiments confirmed that this thermochromic material has excellent thermal stability. Thermogravimetric analysis (TGA) determined 200 °C as the initial temperature of its irreversible thermal decomposition. The material can fully retain its thermochromic activity after continuous exposure to 150 °C for 2 h. Its color-changing performance shows no attenuation under this condition. The material also presents rapid and synchronous color changes. This characteristic makes it suitable for real-time temperature monitoring. In terms of cyclic durability, the material was subjected to 200 repeated heating and cooling cycles from room temperature to 150 °C. It maintained complete structural and functional integrity after these cycles. The thermochromic response speed, color contrast and phase transition temperature accuracy of the material showed no loss [65].

### 2.3. Inorganic Reversible Thermochromic Materials

The primary factors responsible for thermochromism in inorganic compounds include reversible lattice structure changes, reversible alterations in ligand geometry, and reversible desorption and binding of crystalline water [66,67]. These materials comprise metal ion compounds, metal complexes, hydrated inorganic salts, chromates, and their mixtures. With the expansion of temperature-indicating materials, an increasing number of reversible thermochromic substances, such as vanadates, chromates, and tungstates, have been selected for practical applications. Metal ions can be chosen from Group I and II elements, as well as transition metals from Groups IVB, VB, and VIB.

**Figure 6:**
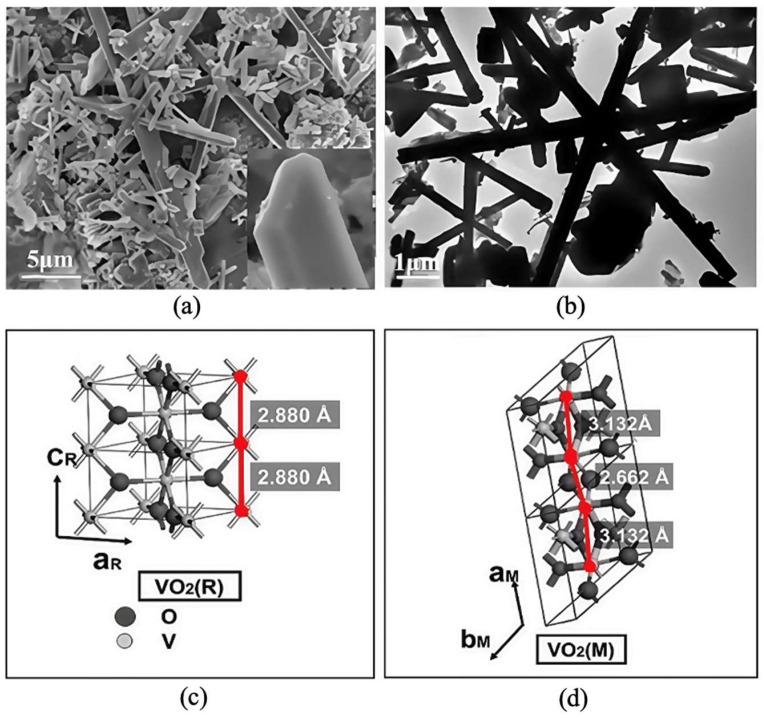
Structure of vanadium dioxide (VO_2_): (**a**) field emission scanning electron microscope (FESEM) image of VO_2_ powder (reused with permission, ref. [71] © 2008, ACS Publications), (**b**) typical low-resolution Transmission Electron Microscope (TEM) image of VO_2_ powder (reused with permission, ref. [71] © 2008, ACS Publications), (**c**) rutile structure (reused with permission, ref. [73] © 2013, Springer Nature), and (**d**) monoclinic structure (reused with permission, ref. [73] © 2013, Springer Nature).

Inorganic reversible thermochromic materials generally contain potentially hazardous elements, including vanadium in VO_2_, as well as chromium, cadmium and lead in certain coordination complexes. These components give rise to prominent biocompatibility and environmental safety concerns. Thus, their applications are mostly concentrated in the field of electrical equipment rather than textile products [23,68,69,70]. However, VO_2_ (Figure 6) represents an exception, demonstrating significant potential for thermochromic smart textiles because of its rapid and reversible phase transition properties [71,72,73]. VO_2_ undergoes a notable transformation from a monoclinic semiconductor phase to a rutile metallic phase upon reaching a specific temperature. Typically, this phase transition occurs around 68 °C, though the exact transition temperature depends on the preparation method [74]. Despite the excellent thermal and chemical stability of such materials, their toxicity issues severely restrict their direct application in smart textiles that come into long-term contact with human skin. Therefore, promising application directions for inorganic thermochromic materials also include industrial technical textiles, special protective clothing with external coatings, and systems with reliable encapsulation to prevent human exposure [75].

### 2.4. Reversible Thermochromic PC Materials

**Figure 7:**
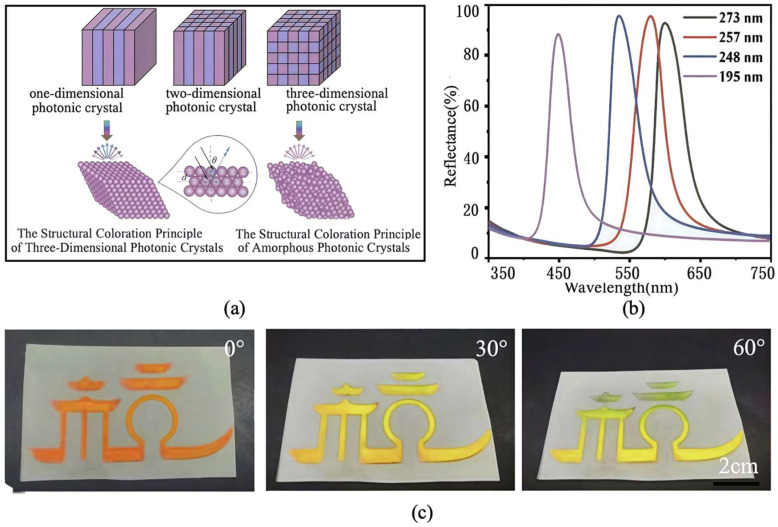
PC materials and structural coloration: (**a**) structural model of PCs and the principle of structural coloration, (**b**) reflection spectrum of polycarbonate structural color fabric assembled from nanospheres of different sizes (reused with permission, ref. [79] © 2024, Small), and (**c**) patterns (reused with permission, ref. [79] © 2024, Small).

Photonic crystals (PCs) exhibit photonic band gaps, also referred to as “photonic forbidden bands,” situated between the energy bands of different materials [76,77,78]. When the photonic band gap lies within the visible light frequency range, visible light of specific wavelengths cannot penetrate the PC. Instead, this light forms coherent diffraction on the crystal surface, resulting in structural color (Figure 7) [79,80]. Temperature variations induce slight structural changes in PCs, such as expansion or contraction of lattice spacing. These modifications alter the band gap width and, consequently, the color of reflected light. Applying this mechanism enables the fabrication of reversible thermochromic structural color textiles. These textiles exhibit high chroma, excellent lightfastness, and are environmentally friendly.

To enhance the functionality of thermochromic PC materials, Xue et al. [81,82,83,84,85,86] developed a simple and rapid in situ polymerization approach. This method enabled the fabrication of dual-responsive PCs with ultra-precise micro-nano hierarchical structures using natural PC templates. The process involved activating reaction sites through deacetylation. This was followed by in situ polymerization of poly(N-isopropylacrylamide-co-acrylic acid) (P(NIPAAM-co-AAc)) on Morpho butterfly wing surfaces. The resulting P(NIPAAM-co-AAc) PCs demonstrated reversible responses to both pH and temperature.

This section covers several reversible thermochromic materials suitable for smart textiles, with their core performance parameters including particle size, thermal stability, color transition characteristics, response speed and cyclic durability summarized in Table 1, which reflects the obvious differences in the basic and application performance of various thermochromic materials at the micro level. Their core performance differences are systematically compared in Table 2, and their key characteristics are briefly summarized as follows: Organic materials have a wide color range and fast response speed, but they lack sufficient stability and wash durability, so they are suitable for non-direct-contact technical textiles like anti-counterfeiting labels. Liquid crystal materials require microencapsulation; they have an adjustable temperature regulation range yet come with high cost and weak color intensity. Thus they are compatible with high-end temperature-monitoring textiles such as medical temperature-sensing fabrics. Inorganic materials show the best stability and durability, but they have a narrow color range and slow response, and they can be used in functional textiles like outdoor protective clothing after surface modification. Photonic crystal materials have vivid structural colors, good biocompatibility, and rapid response; however, their lattice structure is prone to damage during washing, so their wash durability needs improvement, and they are applicable for wearable and fast-response textiles.

Besides the above four types of reversible thermochromic materials, nanostructure modification has become a universal strategy to address the inherent defects of various thermochromic systems such as poor reversibility, slow response speed and insufficient stability [87]. This strategy achieves performance optimization through nanoscale effects and interface regulation. It effectively supplements the intrinsic performance of traditional materials. Nanostructures like nanosheets, nanoparticles and nanofibers can optimize the key properties of materials. An increased specific surface area speeds up the response speed. The size effect enables the regulation of color transition temperature thresholds. It also inhibits material agglomeration and structural degradation, thus improving cycling stability and reversibility. For instance, after polydiacetylene-based nanosheets are embedded into an alginate matrix, the thermochromic reversibility reaches 70% as quantified by RGB image color analysis during the material’s thermal cycling process, and the color saturation is improved at the same time [88]. This regulation strategy is not only applicable to organic systems but also complements the performance optimization of inorganic, liquid crystal and photonic crystal materials. For example, vanadium dioxide nanoparticles can alleviate the agglomeration problem of bulk inorganic materials, and photonic crystal nanospheres can enhance color saturation and response sensitivity [89,90]. In general, nanostructure design enriches the performance regulation approaches of reversible thermochromic materials. It provides more possibilities for their efficient integration into textile substrates.

**Table 1:** Comparison of reversible thermochromic materials.

Material	Size (μm)	Thermal Decomposition Temperature (°C)	Color Transition Temperature Range (°C)	Color Transition Speed	Number of Cycles It Can Withstand	Refs.
Tea polyphenol-based thermochromic dyes	12.8	160	42	——	50	Liu et al. [44]
Thermochromic nanocapsules	0.35–0.8	160	38	——	100	He et al. [48]
Thermochromic microcapsules	0.1–10	158	40.92–45	——	100	Mao et al. [49]
Cyanidin chloride	0.3	160	45	——	——	Khoo et al. [51]
Cholesteric LC Microcapsules	10.9	200	12–42	——	200	Wang et al. [58]
CLCEs	25	150	−31.7–150	——	≥50	Schlafmann et al. [65]
VO_2_ Nanoparticles	<0.1	——	68	——	40	Lu et al. [68]
VO_2_ Microspheres	0.36–0.5	——	67	——	——	De et al. [72]
VO_2_ thin film	0.1	——	68	<1 ps	100	Li et al. [73]
VO_2_ Nanoparticles	0.05–0.06	——	68	——	1000	Peng et al. [75]
Thermochromic PCs Film	0.17–0.33	——	32.0–50.5	54.6 s	2000	Tan et al. [84]
Cholesterol-substituted spiropyran	——	293	≥80	15 min	≥5	Hu et al. [91]
Three-component thermochromic compound	39	150	29.3–33.1	30 s	——	Zhai et al. [92]
Thermochromic Perovskite Microcapsules	18	——	20–70	1 s	50	Yuan et al. [93]
Perovskite hybrid	——	——	70–120	<15 s	50	Cinquino et al. [94]
Thermochromic microcapsules	4–5	300–600	25–35	——	25	Yu et al. [95]
Three-component thermochromic compound	10–20	160	20–35	——	100	Xu et al. [96]
Thermochromic microcapsules	5–14	——	30–80	1 s	30	Yang et al. [97]
Three-component thermochromic compound	10–117	150	32.6–37.5	——	——	Civan et al. [98]
(CEA)_2_CuCl_4_	——	>127	35–120	1 s	——	Zhang et al. [99]

**Table 2:** Comparative evaluation of properties of reversible thermochromic materials.

Evaluation Dimension	Organic Materials	Inorganic Materials	LC Materials	PC Materials
Color Range	Wide, Customizable	Narrow, Limited (Blue/Gray)	Medium, Rainbow/Metallic Luster	Wide, Vivid Structural Color
Stability (Light/Heat/Wash Resistance)	Low (Light, Heat, Wash)	High (Excellent Light, Heat, Wash Resistance After Encapsulation)	Medium (Sensitive to Light and Temperature Extremes) After Encapsulation	Relatively High (Good Lightfastness, Lattice Susceptible to Washing Damage)
Cost	Medium-Low	High	Extremely High	Medium-high
Application	Decorative Textiles (Rapid Response/Rich Colors), Non-direct-contact Technical Textiles	Functional Textiles, Sun-Shading, Outdoor Protective Clothing, Technical Textiles (Prioritizing Stability)	High-End Temp Monitoring, Medical Equipment, Precision Temperature-Sensing Textiles	Wearable Textiles, Rapid-Response Decorative Fabrics, Temperature Indicators, Smart Fabrics (Environmental Adaptation)
Advantages	Vivid Colors, Rapid Response	High Stability, Durable	Temp Control, Adjustable Color-Changing Temperature	High Chroma, Excellent Lightfastness, Environmentally Friendly, Good Biocompatibility, Fast Response
Limitations	Poor Stability, Limited Lifespan	Limited Color Range, Rigidity	High Cost, Environmental Sensitivity	Wash Durability Needs Improvement, Lattice Structure Vulnerable to Repeated Laundering

## 3. Preparation of Reversible Thermochromic Smart Textile

Transforming reversible thermochromic materials into smart textiles necessitates ensuring their secure adhesion to the fabric. To achieve this, a series of intricate printing, dyeing, and finishing techniques are required. A widely adopted method involves the microencapsulation of reversible thermochromic materials. These microcapsules are then applied as core components of printing inks onto the fabric surface through printing and dyeing processes [74,91,92,93,100,101]. Furthermore, various advanced approaches have been developed for the fabrication of reversible thermochromic smart textiles [93,102]. These approaches include fiber preparation techniques, printing and dyeing technologies, and material microencapsulation. The specific implementation methods and research advancements pertaining to these techniques are elaborated upon in the subsequent sections.

### 3.1. Fiber Preparation Technology

Reversible thermochromic textiles are prepared by incorporating reversible thermochromic materials into polymers. The resulting mixture is then fed into the spinning pump to produce fibers with thermochromic properties. This process primary relies on molecular structure changes. Advantages include good color fastness, stability, comfort, a relatively straightforward production process, and excellent oxidation and heat resistance. However, it requires complex equipment and incurs higher production costs.

**Figure 8:**
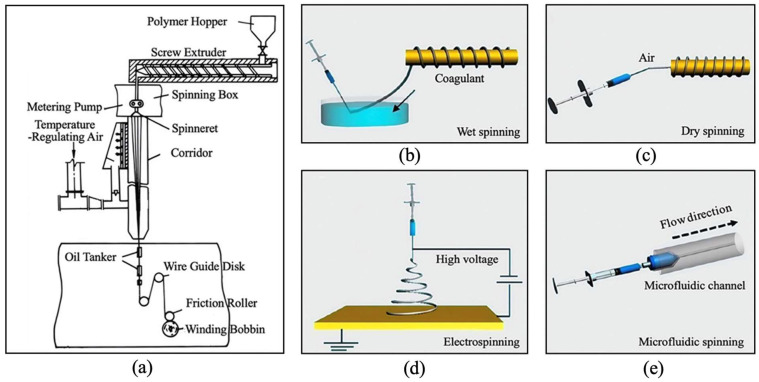
Schematic diagram of different fiber production methods: (**a**) melt spinning, (**b**) wet spinning (reused with permission, ref. [103] © 2019, American Chemical Society, ACS), (**c**) dry spinning (reused with permission, ref. [103] © 2019, American Chemical Society, ACS), (**d**) electrospinning (reused with permission, ref. [103] © 2019, American Chemical Society, ACS), and (**e**) microfluidic spinning (reused with permission, ref. [103] © 2019, American Chemical Society, ACS).

Preparation methods for reversible thermochromic fibers include melt spinning, solution spinning, electrospinning, and microfluidic spinning. Melt spinning is a molding technique that uses polymer melt as the raw material. A melt spinning machine is employed to produce fibers (Figure 8a). Polymers are heated until molten, extruded through spinneret holes, and cooled in air to form fibers. This method includes blend spinning and sheath-core composite spinning. Blend spinning involves melting and mixing high-molecular-weight polymers, such as polyester, with thermochromic polymers for spinning. Alternatively, thermochromic compounds can be compounded with resin carriers to create thermochromic masterbatches, then mixed with polymers for melt spinning. Compared to original fibers, those produced by this method exhibit altered physical properties. These changes include increased fiber diameter and rougher surfaces. However, this method also presents several challenges. These include uneven distribution of thermochromic properties within the fibers and increased viscosity during spinning, which complicates processing. Sheath-core composite spinning prepares temperature-sensitive reversible thermochromic materials compounded with high-molecular-weight polymers as the core layer, while another polymer forms the sheath layer. After melt spinning, temperature-sensitive thermochromic composite fibers are obtained. This technique offers several advantages, including smaller fiber diameters, smooth surfaces, and uniform distribution of thermochromic materials. It also provides lower melt or solution viscosity, which improves processing during spinning.

Solution spinning methods are primarily divided into wet spinning and dry spinning. Wet spinning (Figure 8b), the earlier developed technique, involves extruding a spinning solution into a coagulation bath containing a specific coagulant, which precipitates the solution into solid fibers. Various coagulants have been explored, including organic solvents, acidic or alkaline solutions, inorganic salt solutions, and polymer solutions. However, methanol is toxic and induces rapid coagulation of silk in methanol or ethanol. This rapid coagulation, together with swift structural transformation of the silk protein, can result in insufficient molecular chain orientation and brittle fibers [103]. Therefore, selecting a coagulant should depend on the material, properties, and intended application of the fibers. For instance, Chen et al. [104] used polymer solutions as coagulants. They applied dual-core coaxial wet spinning to fabricate composite fibers. These fibers consisted of a multifunctional conductive hydrogel and a thermochromic elastomer. They featured a core–shell segmented structure. These composite fibers have various applications, including human motion monitoring, body or ambient temperature detection, and color decoration. The diameter of thermochromic fibers produced by wet spinning can be controlled by adjusting the spinneret hole size and the viscosity of the spinning solution. Typically, these fibers exhibit a relatively smooth surface. Dry spinning resembles wet spinning; however, fiber solidification occurs through solvent evaporation or air cooling rather than coagulation in a liquid bath (Figure 8c). Compared to wet spinning, dry spinning offers continuous production and higher spinning speeds, but the equipment is more complex and costly. Furthermore, wet spinning provides superior control over polymer stability and thermochromic properties, making it more suitable for polymers prone to color changes upon heating. Consequently, dry spinning is less commonly used for preparing thermochromic fibers.

Electrospinning primarily utilizes an electric field to generate fibers (Figure 8d). Among its variants, coaxial electrospinning, a specialized form, has been applied for fabricating thermochromic fibers. This method employs a coaxial setup, typically comprising inner and outer electrodes and a nozzle for injecting solution or molten polymer into the electric field. Under a high-voltage electric field, the polymer droplets elongate and solidify into fibers. Coaxial electrospinning enables the production of ultrafine, functional fibers for thermochromic applications; however, challenges remain, including poor fiber uniformity and complex surface morphology. This technique offers greater efficiency and flexibility than traditional spinning methods. However, it requires precise adjustment of two distinct flow rates and solution properties, which complicates process control.

Microfluidic spinning combines the laminar flow characteristics of microfluidics with the rapid prototyping advantages of conventional spinning methods to produce fibers (Figure 8e). In fabricating thermochromic fibers, this approach allows accurate control over fiber diameter, surface morphology, uniformity, viscosity, and draw ratio. It yields fibers with high uniformity and fine structure, potentially enhancing their performance and application prospects. Microfluidic technology involves manipulating microscale fluids within designed microchannels. Its precise and systematic control of individual fluids and interfaces has made it a powerful technique for synthesizing functional materials.

In summary, the core applicable scenarios of different spinning technologies exhibit differences: Melt spinning and microfluidic spinning yield fibers with better wear resistance and wash fastness, suitable for wearable textiles. In contrast, electrospun fibers with poor uniformity show insufficient resistance to sweat and mechanical friction, limiting their use in technical textiles requiring high durability. The detailed comparisons of their core parameters, advantages, limitations, and target applications are presented in Table 3.

### 3.2. Printing and Dyeing Technology

Thermochromic inks are primarily applied to textiles through printing and dyeing techniques. These techniques are used to produce smart textiles that change color in response to temperature variations. Screen printing is commonly employed, using a specially designed mesh to evenly deposit the thermochromic ink on fabric surfaces, which is suitable for creating intricate patterns. Inkjet printing is also frequently utilized due to its high flexibility in fabricating thermochromic smart textiles [105]. Nonetheless, these methods have several limitations. Many printing and dyeing processes involving stimulus-responsive materials that are sensitive to environmental factors such as temperature, humidity, and light. This sensitivity can cause variations in color or performance under adverse conditions, affecting product quality and durability.

**Figure 9:**
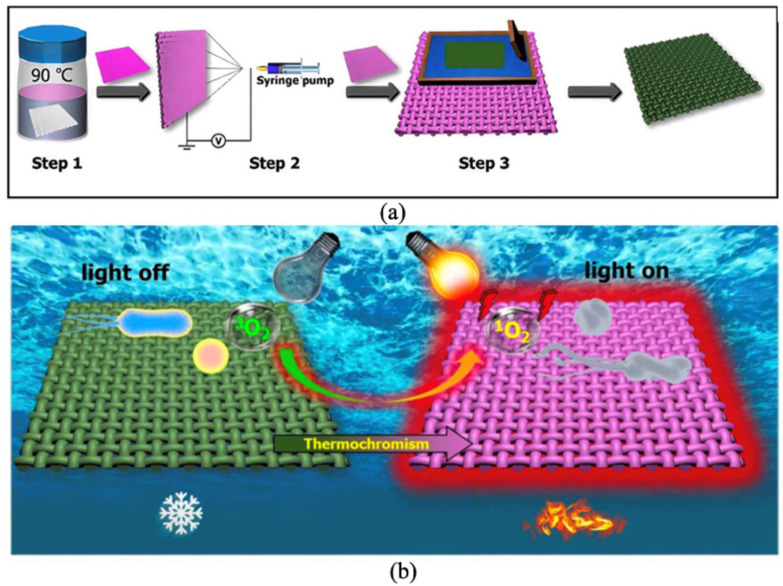
Smart photothermal chromic self-disinfecting textiles: (**a**) preparation process; (**b**) schematic diagram of thermochromic effect (reused with permission, ref. [106] © 2021, ACS Publications).

Nie’s research team [106] successfully developed a photothermal chromic self-disinfecting textile, as shown in Figure 9. This textile was fabricated through in situ growth of PCN-224 metal–organic frameworks (MOFs), electrospraying Ti_3_C_2_ MXene colloids, and screen printing thermochromic dyes. This textile shifts color from dark green to dark red when the temperature exceeds 45 °C. It also exhibits a rapid thermochromic response within one second under 780 nm laser irradiation. However, the use of materials such as PCN-224 MOFs and electrosprayed Ti_3_C_2_ MXene colloids increases production costs due to their high expense. Moreover, temperature changes under intense or prolonged light exposure may reduce wearer comfort. Wang et al. [107,108] developed a thermally activated fluorescent ink composed of multicolor carbon dots (CDs) for computer inkjet printing. The printed patterns exhibit reversible color changes when stimulated by heat or light. However, the formation mechanism of CDs is complex and hinders precise control over their emission wavelengths and thermal activation. As a result, their broad application in printing and dyeing technologies is limited. Future research should focus on improving the stability of thermochromic inks against environmental influences, developing cost-effective materials, optimizing temperature response ranges to enhance comfort, and elucidating the formation mechanisms of CDs. These efforts will improve product quality, reduce cost, and advance the application of smart textiles.

Most thermochromic inks are hydrophilic, as is silk. This similarity complicates achieving strong interfacial adhesion between the two. Conventional industrial silk dyeing involves multiple complex steps, including heating (40–90 °C), soaking (10–50 min), repeated washing and dehydration, and the use of chemical auxiliaries. This process results in substantial wastewater pollution and high energy consumption. Furthermore, reversible thermochromic materials show low reactivity, making them difficult to apply onto silk textiles [107,108]. To overcome these challenges, Wang’s research group [109,110,111,112,113,114] proposed a novel, cost-effective approach combining continuous high-speed winding with dip-coating to enable low-cost, efficient, and scalable silk coloring. In this method, freshly drawn high-tenacity silk fibers from the spinning machine remain partially wet, with water-soluble sericin on the surface acting as a natural high-viscosity adhesive. This significantly improves adhesion between the silk fibers and thermochromic ink. Textiles produced via this technique withstand standard washing tests, exhibiting good color fastness without fading. However, long-term stability and large-scale production still require further verification.

**Figure 10:**
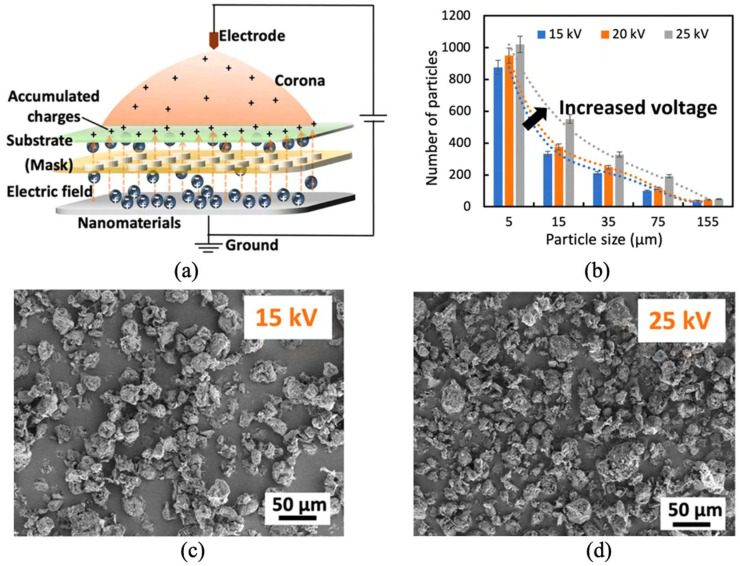
Corona-enabled electrostatic printing: (**a**) crystal structure of high-tenacity reeled silk fibers; (**b**) number of particles with different size ranges attached onto the substrate at corona voltages of 15, 20, and 25 kV; (**c**,**d**) SEM images of graphene particles attached onto the substrate after CEP of 15 and 25 kV (reused with permission, ref. [115] © 2021, ACS Publications).

Additionally, Wang’s team [115] developed a corona-enhanced electrostatic printing method. This technique uses precise corona discharge control to generate a strong electrostatic field on the substrate. The electrostatic field guides the suspension for precise material deposition. The team also modified thermochromic materials and optimized their surface charge distribution, enabling rapid transfer of these materials within milliseconds (Figure 10). This advancement not only speeds up printing but also reduces costs and improves operational safety. Crucially, the method eliminates the need for binders common in traditional approaches, thereby avoiding related losses in material sensitivity during encapsulation.

### 3.3. Material Microencapsulation

Microencapsulation has become a widely adopted technique to improve performance and broaden the application range of reversible thermochromic materials. For example, Matsui Pigment Chemical Industry Co., Ltd., (Kyoto, Japan) utilizes this method to encapsulate reversible thermochromic dyes within microcapsules with a diameter of 3–5 μm. These microcapsules are then firmly attached to fiber products via immersion dyeing. The microencapsulation approach allows textiles to exhibit vibrant colors and highly sensitive color-changing behavior, enabling diverse applications. However, this technique presents several drawbacks. The presence of microcapsules can increase fabric roughness and reduce breathability, resulting in lower wearer comfort. In addition, repeated washing and prolonged use gradually degrade the microcapsule structure, limiting color durability and overall lifespan. Among the various microcapsule fabrication methods, interfacial polymerization and in situ polymerization are commonly employed [91,116,117,118].

**Figure 11:**
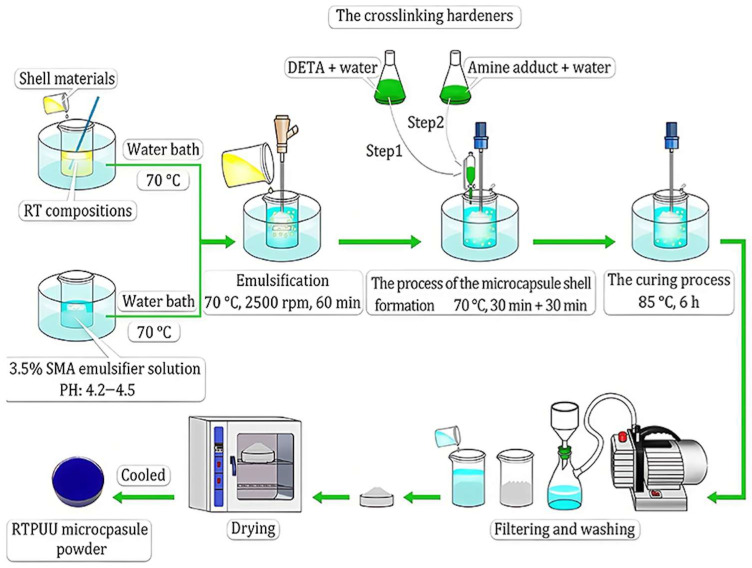
Flowchart for the synthesis of thermochromic microcapsules through interfacial polymerization (reused with permission, ref. [120] © 2023, Multidisciplinary Digital Publishing Institute).

In interfacial polymerization, the core material is usually distributed as the dispersed phase within a continuous phase containing shell material monomers. Additional shell monomers are then added to continue the polymerization. This process forms a polymer film on the core surface, ultimately producing microcapsules. Common shell materials include urea-formaldehyde resin, polyamide, and polyurethane, which form dense microcapsules. However, scaling up interfacial polymerization poses significant challenges. This is because the process is highly sensitive to interfacial conditions that are strongly influenced by mixing parameters. This method offers advantages such as low-temperature operation, rapid polymerization, simple procedures, and good permeability of the shell material. Wang et al. [119] prepared bistable thermochromic microcapsules (BTC-Ms) using interfacial polymerization. They mixed a green thermochromic compound with a MUF prepolymer and carried out interfacial polymerization at controlled pH and temperature, followed by cooling, washing, and drying to obtain microcapsules approximately 1 µm in size. These microcapsules can rapidly and reversibly switch between two color states. They also maintain stability without continuous energy input and can absorb and store heat through phase changes. The bistable color change results from donor–acceptor electron transfer, dynamic hydrogen bonding, and supercooling properties. As a result, the microcapsules show a rapid color response under sunlight at room temperature. Excellent color stability and significant contrast are maintained after 100 lighting cycles. Geng et al. [95,120] successfully prepared formaldehyde-free microcapsules via interfacial polymerization, as shown in Figure 11. Over recent decades, thermochromic microcapsules with melamine-formaldehyde resin shells have been widely commercialized due to low material cost, ease of production, good sealing, and chemical resistance. Nevertheless, melamine-formaldehyde resin releases formaldehyde upon degradation, posing health risks. To address this issue, Liu et al. prepared formaldehyde-free microcapsules using thermochromic compounds as the core and polyurethane-urea (PUU) as the shell. The key to this approach lies in synthesizing the PUU shell. An HDI trimer, diethylenetriamine (DETA), and Epicure 8537-WY-60 (an epoxy resin amine adducts by HEXION, Springfield, OR, USA) were used as crosslinking agents, with CUCAT-U2 (a waterborne polyurethane catalyst from Guangzhou Yourun Corporation, Guangzhou, China) facilitating the reaction. During synthesis, parameters such as emulsification shear rate, core-to-shell mass ratio, and emulsifier concentration were controlled to optimize microcapsule performance. The resulting microcapsule powder exhibited good dispersibility in screen-printing inks and moderate particle size. The microcapsules showed stable thermochromic behavior, excellent thermal stability, UV aging resistance, solvent resistance, and acid–base durability, demonstrating potential for applications such as anti-counterfeiting.

In situ polymerization differs from interfacial polymerization in that the monomers and catalysts for the shell material are present either inside or outside the core droplets during capsule formation. The monomers can dissolve in the continuous phase of the capsule system, whereas the polymers cannot. Consequently, polymerization occurs at the core surface. Monomers continuously deposit and cross-link at this interface, forming a dense polymer shell around the core. Compared to interfacial polymerization, in situ polymerization generally offers easier scalability due to a simpler reaction process and less stringent mixing control requirements. However, achieving uniform monomer distribution within the textile substrate during large-scale production can be challenging, potentially resulting in inconsistent thermochromic performance. Therefore, optimizing monomer impregnation and polymerization processes is essential to ensure consistent product quality. The advantages of in situ polymerization include a broad selection of shell materials, facile control over microcapsule particle size and shell thickness, simple operation, and low cost. Li et al. [121] prepared reversible thermosensitive color-changing microcapsules by in situ polymerization using a composite core of crystal violet lactone, BPA, and tetradecyl alcohol, with urea-formaldehyde resin as the shell. This microencapsulation technique improves the thermal stability and durability of reversible thermochromic materials during use. The microcapsules were formulated into thermochromic inks for printing. Experimental results showed that when microcapsules composed 25% of the ink by mass, the printed samples exhibited optimum adhesion and color difference (ΔE) performance. This technology is applicable in printing and packaging fields such as warning labels, anti-counterfeiting, decoration, and aesthetic enhancement. Wang’s team [122] developed bistable temperature-sensitive color-changing microcapsules (SDBTC-Ms) via in situ polymerization, based on supercooling and spatially restricted electron transfer. This material stably exists in two distinct color states under the same environmental conditions: a colored state (ST I) and a colorless state (ST II). Transition between these states depends on the duration of temperature and light exposure. For instance, after stimulation at 60 °C for 3 min, the material maintains the ST II state for at least 150 h. In contrast, a 1 s stimulus rapidly restores the ST I state. Although the capsules display advantages including bistability, durability, and controllable switching, their complex synthesis and limited color range partially restrict widespread application. Li’s team [96,123,124] synthesized thermochromic microcapsules (TC@MF) by in situ polymerization. The microcapsules feature a thermochromic phase change material (TC-PCM) core and a methylcellulose (MF) shell. This design was aimed at enhancing color variety and photothermal conversion efficiency. These microcapsules were embedded in silicone rubber (SR) to form composites that exhibited high energy storage density, excellent cycling durability, and reversible thermochromic performance. Nevertheless, further investigation is needed to explore the potential application of these capsules in textiles and coatings.

Beyond interfacial and in situ polymerization, several emerging microencapsulation techniques show potential for integrating thermochromic materials into textiles. Ultrasonic impregnation utilizes ultrasonic waves to improve the penetration and uniform distribution of thermochromic compounds within textile fibers. This technique effectively achieves deep and consistent coloration, reduces surface deposition, and enhances wash durability. Pickering emulsions [125], which use solid particles as stabilizers, provide a stable and eco-friendly alternative to traditional surfactant-based emulsions. These emulsions enable the encapsulation of thermochromic dyes, improving their stability and controlled release. Additionally, extrusion and blown film processing have been adapted to produce thermochromic films or coatings that can be directly applied to textiles, offering continuous and scalable manufacturing [126]. Miniemulsion polymerization [127] is a type of emulsion polymerization that forms very small droplets. It facilitates the synthesis of highly stable and uniformly sized microcapsules with precise control over shell composition and morphology. These microcapsules can be incorporated into textile coatings or fibers, serving as a versatile platform for fabricating thermochromic smart textiles.

In summary, the fabrication of reversible thermochromic smart textiles involves several critical technologies. These include fiber preparation, printing and dyeing techniques, and material microencapsulation. Fiber preparation focuses on embedding thermochromic materials within fibers, enhancing fabric durability and comfort. Printing and dyeing transfer thermochromic coatings onto fabric surfaces to achieve patterned color changes. Microencapsulation protects thermochromic materials, improving their stability and resistance to washing. Selecting an appropriate preparation method requires comprehensive evaluation of material properties, application requirements, and cost considerations. This evaluation is necessary to ensure optimal performance and economic efficiency. Future research should prioritize the development of more efficient and environmentally friendly fabrication methods to expand the applications of reversible thermochromic smart textiles.

## 4. Applications of Reversible Thermochromic Smart Textiles

Smart textiles fabricated from reversible thermochromic materials find applications in diverse areas including anti-counterfeiting, decoration and aesthetic enhancement, as well as intelligent management.

### 4.1. Anti-Counterfeiting

An ideal anti-counterfeiting smart fabric integrates reversible thermochromic properties. These properties are unique, dynamic, difficult-to-replicate, stable, and durable. It requires robust anti-counterfeiting performance and durability suitable for practical applications, while providing intuitive and convenient authentication methods [128,129,130,131,132,133]. In this context, reversible thermochromic materials are highly valued in anti-counterfeiting technologies and are widely employed by many apparel brands in concealed embroidered labels and other applications. Li’s team [124] developed an innovative temperature-sensitive ink mainly intended for anti-counterfeiting. By incorporating reversible thermochromic polyurethane (RTPUU) microcapsules into screen-printing ink, they created anti-counterfeiting labels featuring unique reversible thermal memory properties. These labels demonstrate strong color contrast during cooling and heating processes. This behavior significantly improves product identification and security. As a result, the labels offer a novel solution for product anti-counterfeiting. Wang’s team [108] synthesized multicolor CDs with thermally activated fluorescence for multidimensional information encryption. This ink, prepared via a precursor-directed approach, enables full-color emission from 460 nm to 654 nm by precisely adjusting the molecular structure of precursors and synthesis conditions. Furthermore, the fluorescence intensity of the CDs increases with temperature, exhibiting excellent thermochromic behavior. These findings are expected to advance the application of thermochromic CDs in advanced information encryption. They also provide new avenues for developing innovative anti-counterfeiting technologies.

### 4.2. Decoration and Beautification

The application of smart textiles in decoration and beautification demands the integration of multiple functional properties. Aesthetic aspects are critical, including strong visual appeal, trendy design, and personalized customization. In addition, factors such as color fastness, ease of maintenance, cost efficiency, and user comfort must be addressed. Considering these requirements, reversible thermochromic materials present an ideal solution for enhancing the decoration and beautification of smart textiles [134]. For instance, the research team led by Yang [97] combined thermosensitive microcapsules with humidity-sensitive color-changing technology and applied these microcapsules onto fabrics via screen printing. These microcapsules exhibit vivid color, high heat resistance, and solvent durability. As a result, the fabric can respond to simultaneous temperature and humidity variations with notable color-changing performance.

Using reversible thermochromic materials to enhance textile decoration is highly feasible and offers unique creative opportunities. Firstly, these materials can be tailored to designers’ specifications, such as adjusting the color transition temperature and color spectrum, to satisfy the decorative requirements of varied occasions and styles. Moreover, they can be integrated with other textile techniques, including printing and embroidery, thereby further expanding design possibilities. As consumer demand for fashion and individuality grows, reversible thermochromic materials show broad application prospects across sectors including apparel and home textiles. These fabrics not only attract consumer attention but also increase product added value.

### 4.3. Intelligent Management

The development of smart textiles requires evaluating various properties and functional demands [135]. Essential features of smart textiles include environmental sensing, automatic regulation, data processing, communication, and material stability. These features are necessary for functions like temperature regulation, humidity control, and health monitoring. To ensure satisfactory user experience, these fabrics must also meet criteria related to comfort, breathability, and biocompatibility. Additionally, wearability, lightweight design, and material compatibility are critical to accommodate diverse applications. Given these requirements, reversible thermochromic materials are highly appropriate for thermal management. They provide real-time feedback on body or ambient temperature, enabling effective monitoring. Applications include patient caps, protective clothing, and firefighter uniforms [98,136,137,138,139,140,141]. For instance, Zhang’s team [99] developed a novel thermochromic phase change microcapsule with a sandwich-structured shell, serving as an indicator for thermal energy storage and real-time management. This material retains strong thermochromic performance after 500 thermal cycles. Although laboratory results are promising, the durability and reliability of the material in long-term use require further validation. Additionally, the completeness of color change still needs improvement. To extend the temperature indication range, Ge’s team [142] created organic high-temperature reversible thermochromic materials that operate above 100 °C and exhibit rapid color transitions from blue to red. For example, when placed on a 100 °C hot plate, color change begins within one second, and the entire pattern turns red after five seconds. This material also demonstrates good thermal stability and reusability, maintaining color restoration after multiple heating and cooling cycles. It is suitable for thermal indicators in high-temperature environments. However, practical applications demand complex monomer structural designs to regulate the thermochromic temperature threshold, increasing production costs. Furthermore, the specific operating temperature range limits its use in certain scenarios. To improve environmental adaptability and thermal management efficiency, Zheng’s team [143] developed a smart fabric exhibiting temperature-dependent color changes. Through dynamic spectral modulation, the fabric absorbs more solar energy at low temperatures for heating, while reducing solar absorption under high temperatures to achieve cooling.

### 4.4. Challenges

Reversible thermochromic materials have shown progress and potential in smart textiles. However, several challenges still limit their widespread application and commercial viability. A primary issue concerns their durability under real-world use conditions. These materials are sensitive to repeated washing, mechanical abrasion, and prolonged ultraviolet exposure, all of which can impair their color-changing performance and shorten functional lifespan. Although microencapsulation provides some protection, the capsules remain susceptible to damage during laundering or abrasion. This damage may release the thermochromic dye and negatively affect the textile’s appearance and functionality.

Moreover, the restricted color range and possible hysteresis impose further constraints. Organic thermochromic materials offer a broader color spectrum than inorganic ones. However, the available options may still fall short of requirements for precise color matching or intricate designs. Hysteresis, defined as the difference between color transition temperatures during heating and cooling, can reduce temperature indication accuracy and reliability.

Toxicity also represents a significant concern, particularly regarding certain organic dyes and solvents used in thermochromic formulations. Compliance with regulations and consumer safety demands the development of environmentally friendly and biocompatible alternatives. The use of natural dyes such as anthocyanins combined with water-based microencapsulation methods can mitigate some risks. Nevertheless, further studies are necessary to confirm the long-term safety of these materials.

Sustainability constitutes a critical factor as well. The production of some thermochromic materials consumes high energy and depends on nonrenewable resources. Developing sustainable synthesis methods utilizing bio-based or recycled materials is an essential step to reduce environmental impact. Improving the recyclability of thermochromic textiles is also critical. Life cycle assessments can offer valuable insights into the overall sustainability of different thermochromic materials and integration approaches, guiding more responsible design and manufacturing decisions.

## 5. Conclusions and Outlook

In this study, the characteristics, textile integration technologies and practical applications of four mainstream reversible thermochromic materials (organic, liquid crystal, inorganic, photonic crystal) are systematically collated to identify the major technical constraints on their large-scale application in smart textiles, including insufficient wash durability, narrow color expression range, poor environmental stability, and high production costs of partial materials. Also, a summary is presented regarding the progress achieved in exploring nanostructure modification and microencapsulation optimization strategies. This represents a feasible method to improve the performance of thermochromic materials and support their efficient application in textile substrates.

By addressing the above limitations, reversible thermochromic smart textiles can be commercialized in many more industries than traditional clothing. In the field of footwear, it can be used to visually monitor insole temperature and sole load changes. In terms of fashion accessories and sports equipment, such textiles can be transformed into dynamic color-changing products with aesthetic and functional dual attributes. Regarding smart interiors, thermochromic drapes and furniture covers are applicable for intuitive temperature indication and decorative upgrading due to their capability of responding to ambient temperature changes. In terms of technical textiles, these materials are useful for structural overheating early warning in industrial facilities, building structures and transportation equipment, thus enabling visual safety monitoring. Moreover, there are more possibilities in developing the thermochromic materials used in the cultural and creative industry, allowing for technological integration with traditional crafts. It is possible to enhance artistic value by incorporating reversible thermochromic materials into intangible cultural heritage crafts, especially Kesi silk tapestry weaving; ancient Kesi works often adopted precious materials such as gold threads and peacock feathers. Likewise, smart home textiles with dynamic light and shadow effects can be created by integrating thermochromic fibers into Kesi silk tapestry weaving. These textiles not only represent a perfect integration of traditional aesthetic connotation with modern intelligent functionality but also enable the practical application of thermochromic materials and traditional crafts alike.

Regarding the future research and industrial development of reversible thermochromic materials, attention should be paid to three aspects. Firstly, it is essential to construct biologically safe and low-cost thermochromic material systems, widen the adjustable range of color transition temperatures, and enrich chromatic expression. Secondly, it is necessary to optimize textile integration technologies such as microencapsulation and spinning printing, while improving the washing resistance and mechanical stability of thermochromic textiles for compliance with practical application requirements. Lastly, there is a necessity to customize their development to different applications, promote in-depth integration with traditional crafts such as Kesi, and develop multifunctional smart textile products.

Considering the in-depth integration of materials science, textile engineering and intelligent manufacturing, reversible thermochromic smart textiles are expected to be commercialized on a large scale. Apart from promoting the upgrading of the textile industry and the advance of the smart functional materials industry, this also indicates a new pathway to the innovative inheritance and development of traditional intangible cultural heritage crafts, which show promise in being applied in various settings like daily consumption, industrial production, safety monitoring, cultural and creative industries, and others.

## Figures and Tables

**Table 3:** Comparison of different fiber preparation technologies.

Preparation Method	Core Principle	Advantages	Limitations	Application Scenarios
Melt Spinning	Blend thermochromic materials with molten polymers, extrude and cool to form fibers	Good color fastness, high stability, simple process	Complex equipment, uneven property distribution	Wearable smart textiles
Wet Spinning	Extrude spinning solution into coagulation bath for precipitation	Controllable diameter, smooth surface	Toxic coagulants, brittle fibers, long production cycle	Multifunctional composite fibers
Dry Spinning	Solidify fibers via solvent evaporation/air cooling	Continuous production, high speed	Complex equipment, high requirements for the stability of thermochromic materials	Non-direct-contact technical textiles
Electrospinning	Form fibers under high-voltage electric field	Efficient, ultrafine functional fibers	Poor uniformity, complex process control	Temperature-sensing filter materials
Microfluidic Spinning	Combine microfluidic laminar flow with spinning	Precise control over structure, fine fiber structure	High equipment requirements, hard to scale up	Precision temperature-monitoring fabrics

## Data Availability

No new data were created or analyzed in this study. Data sharing is not applicable to this article.
